# Potential merger of ancient lineages in a passerine bird discovered based on evidence from host-specific ectoparasites

**DOI:** 10.1002/ece3.1639

**Published:** 2015-08-18

**Authors:** Nicholas L Block, Steven M Goodman, Shannon J Hackett, John M Bates, Marie J Raherilalao

**Affiliations:** 1 Biology Department, Stonehill College320 Washington Street, Easton, Massachusetts, 02357; 2 Field Museum of Natural History1400 S. Lake Shore Dr., Chicago, Illinois, 60605; 3 Association VahatraBP 3972, Antananarivo (101), Madagascar; 4 Département de Biologie Animale, Université d'AntananarivoBP 906, Antananarivo (101), Madagascar

**Keywords:** Birds, despeciation, ectoparasites, Madagascar, microsatellites

## Abstract

The merger of formerly isolated lineages is hypothesized to occur in vertebrates under certain conditions. However, despite many demonstrated instances of introgression between taxa in secondary contact, examples of lineage mergers are rare. Preliminary mtDNA sequencing of a Malagasy passerine, *Xanthomixis zosterops* (Passeriformes: Bernieridae), indicated a possible instance of merging lineages. We tested the hypothesis that *X*. *zosterops* lineages are merging by comparing mtDNA sequence and microsatellite data, as well as mtDNA sequence data from host-specific feather lice in the genus *Myrsidea* (Phthiraptera: Menoponidae). *Xanthomixis zosterops* comprises four deeply divergent, broadly sympatric, cryptic mtDNA clades that likely began diverging approximately 3.6 million years ago. Despite this level of divergence, the microsatellite data indicate that the *X*. *zosterops* mtDNA clades are virtually panmictic. Three major phylogroups of *Myrsidea* were found, supporting previous allopatry of the *X*. *zosterops* clades. In combination, the datasets from *X*. *zosterops* and its *Myrsidea* document a potential merger of previously allopatric lineages that likely date to the Pliocene. This represents the first report of sympatric apparent hybridization among more than two terrestrial vertebrate lineages. Further, the mtDNA phylogeographic pattern of *X*. *zosterops*, namely the syntopy of more than two deeply divergent cryptic clades, appears to be a novel scenario among vertebrates. We highlight the value of gathering multiple types of data in phylogeographic studies to contribute to the study of vertebrate speciation.

## Introduction

The widespread merger of formerly isolated lineages – sometimes referred to as despeciation or speciation in reverse – is hypothesized to be possible for vertebrates under certain conditions, which often involve human alteration of habitat or introduction of non-native species (Rhymer and Simberloff 1996; Seehausen 2006; Seehausen et al. 2008). Despite many documented instances of introgression between vertebrate taxa in secondary contact, examples of complete lineage mergers, as indicated by total genetic mixing, are rare. The only genetically documented examples of complete lineage mergers in vertebrates, to our knowledge, involve recently diverged lake fish or tanagers (e.g., Taylor et al. 2006; Vonlanthen et al. 2012; Hudson et al. 2013; Kleindorfer et al. 2014). Other likely instances of complete genetic mixing of previously isolated lineages also have been described but have not been analyzed using genetic data (Rhymer and Simberloff 1996; Seehausen et al. 1997). In all of these cases, the lineages involved are evolutionarily young, having diverged since the Pleistocene. Partial or incipient despeciation has been postulated for older vertebrate taxa (e.g., Weckstein et al. 2002; Vallender et al. 2007; Krosby and Rohwer 2009; Webb et al. 2011), but the merger of pre-Holocene lineages has not been documented (for a possible exception, see Hogner et al. 2012). However, despeciation should be possible over these longer timescales because fertile and viable hybrids can be produced between lineages separated for millions of years (Price and Bouvier 2002; Coyne and Orr 2004). In particular, cryptic lineages (those with no significant phenotypic divergence) should be susceptible to merging on a longer timescale due to their reduced pre- and postzygotic barriers to hybridization, such as a lack of morphological or vocal recognition cues. Documenting the scope of this timescale would contribute to the understanding of vertebrate speciation.

If lineages capable of producing fertile offspring come into secondary contact, they may (1) establish sympatry if sufficiently divergent ecologically, (2) maintain parapatry if hybrids have reduced fitness, (3) have one go extinct due to genetic swamping, or (4) create a widening hybrid swarm and eventually merge after a sufficient amount of time (Liou and Price 1994; Servedio and Noor 2003). Here, we refer to either of the last two scenarios as despeciation. Just as lineages splitting in the process of speciation are not always considered species, we assert that lineages merging in the process of despeciation do not necessarily have to be species.

Completed despeciation is difficult to document, particularly between cryptic taxa, because most lineage-specific nuclear characters will have been obscured by widespread hybridization, although high levels of nuclear heterozygosity and/or polymorphism may provide suggestive evidence. However, due to its lack of recombination, mitochondrial DNA (mtDNA) may prove more useful in detecting possible lineage mergers because significant sympatric genetic structure would likely remain long after the lineages merge (e.g., Weckstein et al. 2002; Hogner et al. 2012). Sympatric structure in mtDNA without congruent nuclear structure does not immediately imply despeciation, as other phenomena can produce similar patterns, such as retained ancestral polymorphism (e.g., Mims et al. 2010), the presence of nuclear copies of mitochondrial DNA (numts) (e.g., Sorenson and Quinn 1998), selection on mtDNA (e.g., Ballard and Whitlock 2004), mitochondrial capture (e.g., Weckstein et al. 2001), or brood parasitism causing host-specific maternal lineages (e.g., Spottiswoode et al. 2011). Of these phenomena, ancestral polymorphism and numts are the most likely to mirror the pattern produced by a lineage merger, namely widespread sympatry of divergent mtDNA clades with no corresponding structure in nuclear markers. Careful research design and sequence analysis can eliminate the possibility of numts (Sorenson and Quinn 1998), but retained ancestral polymorphism (whether due to balancing selection or incomplete lineage sorting) is much more difficult to separate from an ongoing merger of cryptic lineages.

In the absence of phenotypic differences associated with mtDNA lineages or genomic level nuclear DNA data that could exclude a scenario of retained ancestral polymorphism, it is possible to use host-specific parasites as proxies for host traits (e.g., Whiteman and Parker 2005; Nieberding and Olivieri 2007) to provide an independent test of the ancestral polymorphism hypothesis. If host sympatric mtDNA structure were due to ancestral polymorphism retained in a broadly panmictic population, then the host-specific parasites likely would not exhibit similarly deep genetic structure. The smaller effective population sizes and shorter generation times of the parasites would result in more rapid lineage sorting than their hosts (Rannala and Michalakis 2003; Nieberding and Olivieri 2007), reducing ancestral polymorphism. Therefore, if host sympatric mtDNA structure were due to a scenario of despeciation, distinct parasite lineages also would be present due to codiversification with the previously isolated host lineages.

Our preliminary mtDNA sequencing of *Xanthomixis zosterops* (Sharpe, 1875), a forest-dependent Malagasy passerine belonging to the endemic family Bernieridae (Cibois et al. 2010), showed the presence of multiple sympatric, cryptic, deeply divergent clades that did not sort strongly by geography or recognized subspecies (Salomonsen 1934; Clements et al. 2014), indicating possible despeciation. We test the hypothesis that the *X*. *zosterops* clades are merging by examining DNA sequence data from mitochondrial and nuclear markers across much of its known range, as well as sequence data from feather lice (*Myrsidea*, Phthiraptera). *Myrsidea* have been shown to exhibit strong host specificity, and multiple host-specific *Myrsidea* have not been documented on a single host species before (e.g., Price and Dalgleish 2006; Bueter et al. 2009; Štefka et al. 2011). Thus, *Myrsidea* provide an appropriate means to test for host despeciation, as described in the previous paragraph, because their evolutionary history accurately mirrors the history of their hosts.

If *X*. *zosterops* represents a merger of ancient cryptic lineages, we expect widespread sympatry of the *X*. *zosterops* mtDNA clades, no significant nuclear structure within *X*. *zosterops* (due to widespread gene flow among the mtDNA clades), and the presence of distinct *Myrsidea* clades (due to codiversification with the previously isolated *X*. *zosterops* clades).

## Materials and Methods

### Taxon sampling

We obtained DNA from 117 *X*. *zosterops* individuals, using 28 feather and 89 muscle tissue samples (Table S3). All samples were obtained from material accessioned at the Field Museum of Natural History (FMNH) or the Université d'Antananarivo, Département de Biologie Animale (UADBA). Sample localities span virtually the entire known range of *X*. *zosterops*, although our coverage is sparse in the lower elevations of the central east.

Bird DNA extraction methods varied, depending on the tissue type. Muscle samples were extracted using a DNEasy® Blood & Tissue Kit (Qiagen, Valencia, CA), following manufacturer's protocols. For feather samples, we used a QIAmp® DNA Micro Kit (Qiagen), modifying the manufacturer protocol to add 30 *μ*L 1M dithiothreitol (DTT) during the initial digestion step.

Single lice from 18 host individuals were extracted using a QIAmp® DNA Micro Kit, and a voucher was retained following the extraction protocol. Most *Myrsidea* were collected in the field from live birds using the flea powder dusting method of Walther and Clayton (1997), but the *M*. *yoshizawai* (Price & Johnson 2006) individual was collected from a bird specimen using ethyl acetate fumigation. *Myrsidea* DNA voucher specimens were deposited in the FMNH Insect Collection (Table S4).

### DNA sequencing

For almost all *X*. *zosterops* samples, we obtained sequence from two mtDNA genes. For 110 samples, we sequenced 395 base pairs (bp) of the mitochondrial NADH dehydrogenase subunit 3 gene (ND3) and its flanking tRNA genes. For 115 samples, we sequenced 911 bp of the cytochrome *b* (cyt-*b*) gene. Not all samples were sequenced for both cyt-*b* and ND3 due to PCR difficulty or degraded DNA. Additionally, to help resolve the final tree topology, we sequenced 673 bp of the mtDNA ATP synthase F0 subunit 6 gene (ATP6) for 53 samples.

For all *Myrsidea* samples, we sequenced a 379-bp piece of the mitochondrial cytochrome *c* oxidase I (COI) gene.

The PCR, purification, and sequencing methods for all genes followed standard protocols with a few variations dependent on PCR quality (Appendix S1). All sequences were visualized and aligned in BioEdit v7.0.9 (Hall 1999). For mitochondrial genes, sequences were checked for stop codons or distinct double peaks that would indicate the presence of nuclear pseudogenes.

### Phylogenetic analysis of sequence data

We constructed a phylogeny of all unique *X*. *zosterops* mtDNA haplotypes using standard methods of maximum parsimony (MP), maximum likelihood (ML), and Bayesian inference (BI) (Appendix S1). The two sister taxa of *X*. *zosterops*, *Xanthomixis cinereiceps* (Sharpe, 1881) and *Xanthomixis apperti* (Colston, 1972) (Cibois et al. 2001), were used as outgroups. For ML and BI analyses, partitioning schemes and best-fit models were simultaneously determined in the program Kakusan4 (Tanabe 2011), using a corrected Akaike information criterion (AIC) (Akaike 1974).

To make direct comparisons with avian molecular clock studies, we calculated average cyt-*b* divergences among and within *X*. *zosterops* mtDNA clades for p-distances and corrected distances, following the correction methods of Weir and Schluter (2008) and using a family-level dataset to obtain model parameters. Analysis of the *Myrsidea* data followed the above methods, except the *Myrsidea* taxon from *Crossleyia xanthophrys* (Sharpe, 1875), another member of the Bernieridae, was used as the outgroup.

### Population genetics and historical demography (mtDNA)

For each *X*. *zosterops* mtDNA clade, we calculated standard diversity indices – haplotype diversity (H) (Nei and Tajima 1981), nucleotide diversity (*π*) (Nei and Li 1979), and the Watterson estimator (*θ*_w_) (Watterson 1975) – for a dataset combining ND3 and cyt-*b*. To test for any recent demographic changes within clades, we calculated the Fu's *F*_*S*_ (Fu 1997) and *R*_2_ (Ramos-Onsins and Rozas 2001) statistics based on the number of segregating sites. We tested for selection on the mtDNA markers using the McDonald–Kreitman test (McDonald and Kreitman 1991). All calculations and tests were performed in DnaSP 5.10 (Librado and Rozas 2009).

### Microsatellite genotyping and analysis

To gain a more complete understanding of population genetic structure within *X*. *zosterops*, we developed 10 variable microsatellite loci and genotyped 104 *X*. *zosterops* individuals using standard protocols (Appendix S1). Using Arlequin 3.5 (Excoffier and Lischer 2010), we tested each of the 10 microsatellite loci for departure from Hardy–Weinberg equilibrium and tested for linkage disequilibrium between loci pairs. Loci not in Hardy–Weinberg equilibrium were not included in the final analyses. For each locus, we also calculated gene diversity and allelic richness in FSTAT 2.9.3 (Goudet 2001).

To determine the level of genetic differentiation in microsatellites among the *X*. *zosterops* mtDNA clades, we calculated three differentiation statistics and conducted an AMOVA. Pairwise *F*_ST_ values were calculated in Arlequin, with *P*-values determined by 10,000 permutations. We used GenAlEx 6.41 (Peakall and Smouse 2006) to compute pairwise *R*_ST_ (Slatkin 1995) values, running 999 permutations to determine significance levels. As *F*_ST_ and *R*_ST_ are not true measures of differentiation, particularly when genetic diversity and differentiation are high (Jost 2008), we also used SMOGD 1.2.5 (Crawford 2010) to generate pairwise Jost's D (Jost 2008) values.

Determining accurate population clustering patterns, particularly in the presence of strong admixture, is a nontrivial exercise, and different approaches can produce different results (Gao et al. 2011). Thus, we implemented a variety of methods to infer possible structure among the microsatellite data. First, we used the Bayesian clustering approach implemented in Structure 2.3.3 (Pritchard et al. 2000; Falush et al. 2007; Hubisz et al. 2009), following standard methods for *K *=* *1–8 (Appendix S1). The optimum value of *K* was determined by examination of lnP(D) and *α* across the runs, as well as the Δ*K* statistic (Evanno et al. 2005). We also used the *H′* statistic from CLUMPP 1.1.2 (Jakobsson and Rosenberg 2007), which measures similarity of runs, when determining the optimum *K* value. Second, we used standard methods (Appendix S1) in Structurama (Huelsenbeck et al. 2011) to compare its calculated optimum *K* to the results from Structure. Third, we analyzed the data in TESS 2.3.1 (Chen et al. 2007; Durand et al. 2009), which implements the deviance information criterion (DIC) to help users decide the optimum *K*. TESS also incorporates geographic distance information among samples via a modifiable spatial network.

## Results

### *Xanthomixis zosterops* mitochondrial phylogeography

**Figure 1:**
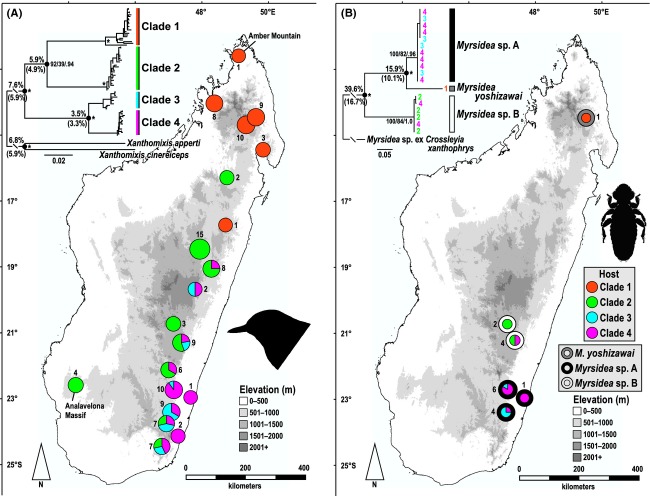
Phylograms and sample distribution maps of *X*. *zosterops* and its associated *Myrsidea*. (A) ML phylogram of *X*. *zosterops* from 1979 bp of mtDNA (ND3, cyt-*b*, and ATP6), collapsed to unique haplotypes. Nodes labeled with cyt-*b* corrected and p-distance (in parentheses) divergences and with nodal support (MP/ML/PP, *=100/100/1.0). Map shows distribution of *X*. *zosterops* mtDNA clades, with pie charts showing proportion of each clade among samples from a given area. Numbers next to pie charts are sample sizes. (B) ML phylogram of all *Myrsidea* feather lice collected from *X*. *zosterops*, based on 379 bp of mtDNA (COI). Numbers at tips represent the *X*. *zosterops* clade from which the individual was collected. Nodes labeled with corrected and p-distance (in parentheses) divergences and with nodal support (MP/ML/PP, *=100/100/1.0). Map shows geographic distribution of *Myrsidea* samples. Internal pie charts represent proportion of *Myrsidea* collected from each of the four *X*. *zosterops* mtDNA clades. Numbers next to pie charts are sample sizes.

The topology of the *X*. *zosterops* mtDNA phylogeny reveals four distinct clades with deep divergences (Fig.1A); average corrected cyt-*b* divergences at the three major nodes are 3.5% (3.3% p-distance), 5.9% (4.9%), and 7.6% (5.9%). Using the average passerine molecular clock estimate of ∼2.1% per million years (Weir and Schluter 2008), cladogenesis beginning approximately 3.6 million years ago (Ma) is consistent with these data. As a contextual reference, *X*. *cinereiceps* and *X*. *apperti* – the two allopatric, phenotypically distinct congenerics of *X*. *zosterops* – are 6.8% (5.9% p-distance) divergent from each other based on cyt-*b*. This level of divergence within *X*. *zosterops* is unexpected because geographic ranges of the clades largely overlap. Clades 2–4 are widely syntopic in the southern portion of the eastern humid forest, whereas clades 1–2 are at least latitudinally sympatric in the central north portion of this zone (Fig.1A). Syntopy and extent of range overlap of clades 1–2 are unknown due to sparse sampling in this central north region. The geographic distributions of the clades do not match those of the four recognized subspecies, as is common for birds (Zink 2004). Clade 1 roughly corresponds to *X*. *z*. *andapae* (Salomonsen 1934), but it also includes the distinctly paler *X*. *z*. *fulvescens* (Delacour, 1931) from Amber Mountain. The widespread Clade 2 is found within the described range of three subspecies [*X*. *z*. *andapae*, *X*. *z*. *ankafanae* (Salomonsen 1934), and *X*. *z*. *zosterops*], Clade 3 in one (*X*. *z*. *zosterops*), and Clade 4 in two (*X*. *z*. *ankafanae* and *X*. *z*. *zosterops*). The mtDNA sequences lack stop codons and indels within coding regions and double peaks in sequence chromatograms, and phylogenetic results of all three mtDNA markers are congruent when analyzed separately, both suggesting that the *X*. *zosterops* phylogeography is not the result of numts.

### Population genetics and historical demography (mtDNA) of *Xanthomixis zosterops*

The combined mtDNA data show that all four *X*. *zosterops* clades exhibit high levels of genetic diversity, with Clade 4 being slightly less diverse than the other three clades (Table1). These results suggest that no clade has experienced a recent population bottleneck. Fu's *F*_*S*_ and *R*_2_ values are significant in clades 1, 2, and 4, whereas Clade 3 values are near significant (Table1). These results support demographic expansion among the clades, with the strongest signal present in Clade 2. The *P*-values of the McDonald–Kreitman test were not significant for any pair of clades, showing that selection on mtDNA likely has not affected the phylogeography of *X*. *zosterops*.

**Table 1:** Genetic diversity statistics and population expansion tests for *X*. *zosterops* mtDNA clades, based on combined sequence data from cyt-*b* and ND3 (1306 bp)

mtDNA Clade	*N*	H	*π*	*θ*_w_	Fu's *F*_*S*_	*R*_2_
1	32	0.919	0.00512	0.01121	**−6.373**	**0.0631**
2	36	0.949	0.00265	0.00488	**−17.155**	**0.0535**
3	10	0.978	0.00441	0.00489	**−**3.146	0.1176
4	23	0.889	0.00198	0.00295	**−5.339**	**0.0766**

H, haplotype diversity; *π,* nucleotide diversity; *θ*_w_, Watterson estimator. Values of significant population expansion tests (*P *<* *0.02 for Fu's *F*_*S*_, *P *<* *0.05 for *R*_2_) are in bold.

### Microsatellite analysis

**Figure 2:**
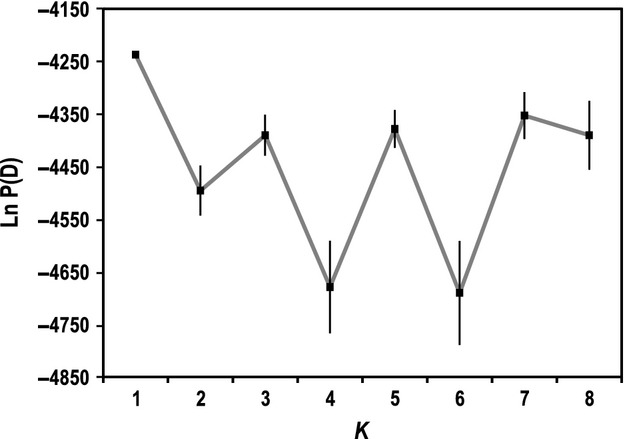
The average likelihood and standard error for each *K* calculated over 10 Structure runs.

**Figure 3:**
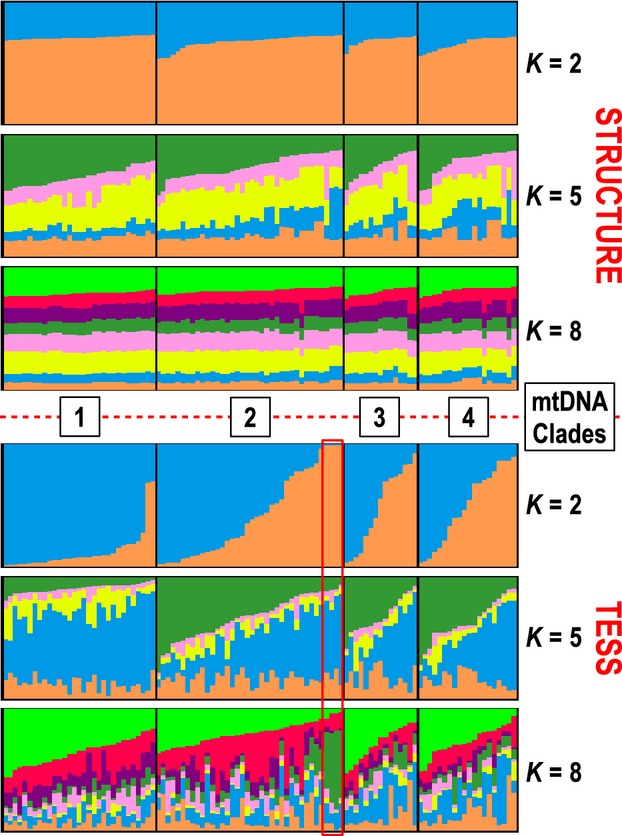
*Xanthomixis zosterops* population clustering analyses from Structure and TESS, based on eight microsatellite loci. Samples are organized by mtDNA clade and clustering scores, and the Analavelona Forest samples are highlighted in red.

Structure analyses show that the number of population clusters (*K*) that is optimal (i.e., has the highest lnP(D) value) is *K *=* *1, which implies panmixia among the mtDNA clades (Fig.2). Individual population assignments from TESS analyses are more varied than those from Structure, but the results do not strongly contradict *K *=* *1 (TESS cannot analyze *K *=* *1). The TESS runs for *K *=* *2 have the highest *H′* value in CLUMPP (Fig. S2), but the value is not very near 1.0, indicating relatively weak support for *K *=* *2. However, unlike the near-symmetric individual assignments from Structure, the TESS plot for *K *=* *2 shows wide variation in assignment proportions (Fig.3). Population assignment proportions are skewed in one direction within Clade 1, whereas the other three clades have a relatively even mix. Another notable result is that the four individuals from Analavelona Massif, an isolated montane humid forest in southwestern Madagascar (Fig.1) that is surrounded by dry forest, are the only samples with near 100% population assignment.

In Structure, the a priori expected result of *K *=* *4 has the second lowest value of lnP(D). The *K *=* *8 runs have the highest *H′* value, but this is due to convergence on near-symmetric population assignment across all samples (Fig.3), which is associated with a lack of population structure and *K *=* *1 (Pritchard et al. 2000). The Δ*K* statistic, which cannot evaluate *K *=* *1, peaks at *K *=* *5 (Fig. S1). However, the clustering of *K *=* *5 does not reflect any significant microsatellite population structure that correlates with mtDNA structure or geography, and the *H′* value for K = 5 is relatively low, further supporting *K *=* *1 for the microsatellite data (Fig.3). The Structurama analysis, genetic differentiation tests, and AMOVA all support *K *=* *1 as well (SI Results).

### *Myrsidea* mitochondrial phylogeography

As with *X*. *zosterops*, the *Myrsidea* mtDNA phylogeny contains significant structure, with three major phylogroups recovered (Fig.1B). In contrast to their host *X*. *zosterops* mtDNA clades, however, the *Myrsidea* phylogroups appear to be allopatric, although the sampling is limited. These results support the ancestral presence of at least three distinct *X*. *zosterops* populations. The model-corrected average divergences at the two major nodes of the *Myrsidea* phylogeny are 15.9% (10.1% p-distance) and 39.6% (16.7%). Although the three phylogroups are not readily distinguishable phenotypically (M. Valim, pers. comm.), these divergence levels are consistent with other species-level divergences among *Myrsidea* (Bueter et al. 2009).

## Discussion

We provide evidence for widespread nuclear admixture of four deeply divergent and previously isolated mtDNA lineages in *X*. *zosterops*, corresponding to the hypothesis of lineage merger. This represents the first report of sympatric apparent hybridization among more than two terrestrial vertebrate lineages. Further, the mtDNA phylogeographic pattern of *X*. *zosterops*, namely the syntopy of more than two deeply divergent cryptic clades, appears to be a novel scenario among vertebrates.

This widespread sympatry of divergent, interbreeding mtDNA lineages might arise for a number of reasons, such as secondary contact of previously allopatric lineages, ancestral polymorphism (e.g., Mims et al. 2010), numts (e.g., Sorenson and Quinn 1998), selection on mtDNA (e.g., Ballard and Whitlock 2004), mitochondrial capture (e.g., Weckstein et al. 2001), or brood parasitism (e.g., Spottiswoode et al. 2011). The results of the McDonald–Kreitman test are not statistically significant, suggesting that selection has not played an important role in the mtDNA variation of *X*. *zosterops*. A scenario of mitochondrial capture would show paraphyly of *X*. *zosterops* with another species or nuclear structure among the *X*. *zosterops* mtDNA clades. Thus, the monophyly of *X*. *zosterops* and lack of nuclear structure among the mtDNA clades reject the mitochondrial capture hypothesis. *Xanthomixis zosterops* is not a brood parasite, eliminating the possibility that the mtDNA clades represent host-specific matrilineal lineages. Although the unique phylogeographic pattern in *X*. *zosterops* is suggestive of unusually divergent numts, this hypothesis is rejected by characteristics of the mtDNA sequences.

The *Myrsidea* chewing lice phylogeny resolves which of the two remaining and most likely explanations – retained ancestral polymorphism and widespread secondary contact – best explains the *X*. *zosterops* scenario. If the widespread sympatry of the divergent *X*. *zosterops* clades were due to ancestral polymorphism in a panmictic population, *Myrsidea* would be freely transmitted among the interbreeding members of the *X*. *zosterops* clades. With this scenario, gene flow among the lice would not be impeded, and notable divergences would be unlikely to arise. However, if widespread secondary contact explains the current phylogeographic patterns of *X*. *zosterops*, divergence in allopatry would result in multiple clades of host-specific *Myrsidea* inhabiting *X*. *zosterops*. Therefore, the three distinct phylogroups of *Myrsidea* provide strong evidence for the previous allopatry of *X*. *zosterops* clades. As sampled, these three *Myrsidea* phylogroups are specific to *X*. *zosterops* and distinct from *Myrsidea* found on other members of Bernieridae (Block 2012) – although *X*. *apperti* has yet to be sampled for lice – further supporting a scenario of codiversification (as opposed to host switching). Although our *Myrsidea* sample size is small due to the difficulty of obtaining large numbers in a short amount of time in Madagascar, a larger sample size would not alter the primary result of distinct phylogroups unique to *X*. *zosterops*. This case illustrates that host-specific ectoparasites can be powerful tools for confirming secondary contact among host lineages and for separating this scenario from other evolutionary phenomena.

The *Myrsidea* data also provide useful information in trying to reconstruct the previous allopatric ranges of the *X*. *zosterops* clades. Although possibly sympatric with Clade 2 at the southern end of its current range, Clade 1 is largely allopatric in the far north of the island, suggesting that it was latitudinally separated from the other clades (Fig.1A). This conclusion is supported by a unique *Myrsidea* phylogroup found in the north (Fig.1B), although this support is tempered by the limited *Myrsidea* sampling in the region. Untangling the geography history of clades 2–4, given their widespread syntopy, is more difficult. Four lines of evidence support the hypothesis that Clade 2 and clades 3/4 were separated altitudinally, with Clade 2 being found at higher elevations.

First, the two phylogroups of *Myrsidea* found on these three clades appear to be allopatric, despite the sympatry of their hosts. *Myrsidea* sp. A is present on individuals of clades 3–4 and only at lower elevations (60 m, 590 m, and 630 m), whereas *Myrsidea* sp. B has been found on individuals of clades 2 and 4 and only at higher elevations (1150 m and 1710 m). These elevational differences between the *Myrsidea* phylogroups may mirror historical differences between the *X*. *zosterops* clades before current secondary contact. However, the low level of *Myrsidea* sampling limits the strength of this argument; we cannot rule out the possibility that larger *Myrsidea* sample sizes may show some sympatry of the phylogroups.

**Figure 4:**
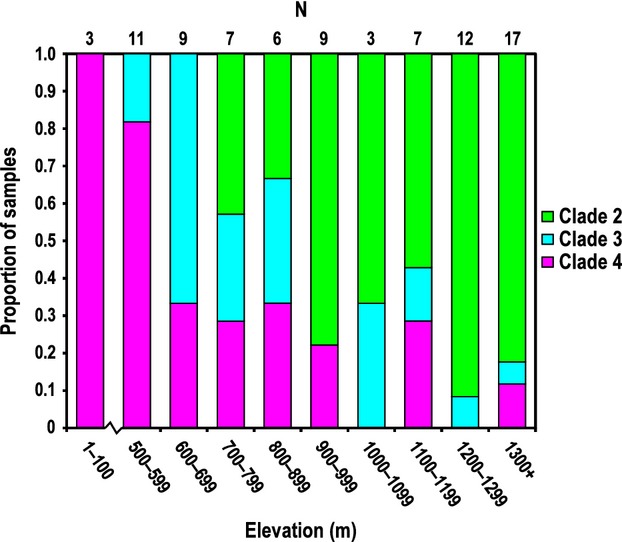
The frequency of each of the three widely sympatric *X*. *zosterops* clades in a given elevational range. Sample sizes for each elevational zone are indicated on the upper *x*-axis.

Second, we examined the frequency of clades 2–4 at given elevational ranges to search for patterns that might be informative about historical elevational ranges of the clades (Fig.4). The results support previous altitudinal separation between Clade 2 and clades 3/4, with a steady decline in the frequency of clades 3/4 (and increase in Clade 2) as elevation increases. These results might be expected following a breakdown of former isolating factors and subsequent dispersal of females across previous elevational limits.

Third, evidence from another species in the Bernieridae, *Bernieria madagascariensis* (Gmelin, 1789), further supports previous allopatric clade ranges separated altitudinally (Block 2012). *Bernieria* comprises three cryptic phylogroups, similar to *X*. *zosterops* but more genetically divergent. Unlike *X*. *zosterops*, these three lineages remain parapatric. Two sister phylogroups are separated latitudinally, with one in the far north of Madagascar and the other at elevations below ∼1000 m in the southeast and central east. These two taxa are then sister to another phylogroup that is found in the east only at elevations above 1000 m. This pattern strongly mirrors the phylogeography of the *Myrsidea* found on *X*. *zosterops* and may reflect similar aspects of parallel historical phylogeography of these two species of Bernieridae.

Finally, in Madagascar, lowland and montane humid forests represent extremes along an elevational gradient (Moat and Smith 2007). Montane forests typically are cooler and more consistently humid, receive more rainfall, and have reduced canopy heights compared to lowland forests. Species turnover between the two habitats across different taxa is often high, with the zone of transition usually around 800–1000 m (e.g., Raxworthy and Nussbaum 1994; Olson et al. 2004; Vieites et al. 2006).

These four lines of evidence – *Myrsidea* phylogroups, current clade elevational frequencies, *Bernieria* phylogeography, and elevational habitat differences – provide support for the three oldest *X*. *zosterops* mtDNA clades occupying previously allopatric or parapatric ranges, with Clade 1 (and *M*. *yoshizawai*) in the far north, Clade 2 (and *Myrsidea* sp. B) in montane forests throughout the east, and clades 3/4 (and *Myrsidea* sp. A) in lowland forests in the central east and southeast. Based on the available evidence, we cannot confidently comment on the possible mechanism of diversification between clades 3 and 4.

Widespread sympatry of cryptic mtDNA lineages due to secondary contact, such as appears to be the case in *X*. *zosterops*, is likely to lead to despeciation via lineage merger or genetic swamping in the absence of genetic incompatibilities. The lack of prezygotic reproductive barriers (e.g., plumage differences) and postzygotic barriers (e.g., hybrid inviability) would lead to widespread hybridization and a loss of nuclear genetic structure. The microsatellite data support the hypothesis that *X*. *zosterops* at least partially demonstrates a case of despeciation occurring in this way.

Results from the microsatellite analyses indicate that the *X*. *zosterops* mtDNA clades exhibit no strongly congruent nuclear structure and are now significantly admixed in widespread secondary contact, despite as much as 3.6 million years of divergence (based on mtDNA). Although evidence from previous studies suggests that initial microsatellite results can be supported or strengthened with further sequencing data (Dierickx et al. 2015), we recognize that the microsatellites sequenced for this study represent only a very small portion of the genome and that our conclusions are limited by this low genetic coverage. We plan to investigate *X*. *zosterops* using genomic-level sequencing in the future.

The *X*. *zosterops* scenario potentially represents the first demonstrated merger of more than two lineages of vertebrates and possibly the first of lineages that diverged pre-Holocene. Although 3.6 million years of divergence is more than many noncryptic sister species of passerines, our data are in agreement with studies showing that the successful establishment of widespread sympatry between cryptic bird species may take a significantly larger amount of time (Price 2010). For example, within two genera of Old World leaf warblers (*Phylloscopus* and *Seicercus*), the youngest pair of widely sympatric, near-cryptic species diverged approximately 6 Ma (Päckert et al. 2004; Price 2010).

The results support the merger of at least *X*. *zosterops* clades 2–4 via dispersal of multiple clades and subsequent widespread hybridization, rather than a case of genetic swamping by one primary clade. If the *X*. *zosterops* scenario reflected an ancient genetic wake left by a dominant dispersing clade that swamped out the other lineages, this clade likely would be the only one with a genetic signal of demographic expansion (e.g., Krosby and Rohwer 2009). Additionally, populations in the region from which the dispersing clade originated would remain genetically “pure,” resulting in microsatellite clustering of *K *≥* *2. However, the significant evidence for demographic expansion in three *X*. *zosterops* clades, the microsatellite results supporting *K *=* *1, and the elevational distribution data in Figure4 support the merger being the result of multidirectional dispersal and a widening hybrid swarm of at least clades 2–4. The TESS results suggest that gene flow involving Clade 1 may be less symmetric, with more gene flow out of Clade 1 than vice versa.

Dispersal of nuclear markers has been more pronounced than mtDNA markers. The lack of strong geographic structure in microsatellites is in contrast to the incomplete sympatry of the mtDNA clades, particularly Clade 1. Although the TESS analysis shows some structure for Clade 1 if *K *=* *2 (Fig.3), *K *=* *1 is still the optimal solution. This apparent geographic conflict of mtDNA and nuclear data has two likely explanations: male-biased dispersal and/or Haldane's rule, which states that the heterogametic sex among hybrids is more likely to be sterile (Haldane 1922). Male-biased dispersal is well-documented as a reason for homogenizing gene flow among distinct mtDNA lineages within species (e.g., Melnick and Hoelzer 1992; Gibbs et al. 2000), although female-biased dispersal seems to predict greater nuclear than mtDNA introgression between species (Petit and Excoffier 2009). Explanations for male-biased dispersal vary, but mating system and resource control are the two primary factors invoked (Greenwood 1980; Clarke et al. 1997; Wolff and Plissner 1998). Polygynous species are more likely to exhibit male-biased dispersal, but *X*. *zosterops* is likely monogamous, based on field observations at the nest. Additionally, the sex that more strongly defends resources and territories is likely to be philopatric, whereas the other sex has higher dispersal, but resource defense in *X*. *zosterops* has not been studied. Although the lack of specific knowledge regarding dispersal in *X*. *zosterops* makes it difficult to directly eliminate male-biased dispersal as a factor in the incongruency between mtDNA and nuclear DNA, we believe the *Myrsidea* data indirectly eliminate the male-biased dispersal scenario. Although males would not spread mtDNA, leading to mtDNA structure in a species if females were sedentary, they would still spread parasites among populations. If dispersal were occurring, even if male-biased, *Myrsidea* populations would not become isolated enough to produce the strong genetic structure illustrated by those found on *X*. *zosterops*.

Male-biased dispersal would explain the discord between the mtDNA phylogeography and the microsatellite results, but the effects of Haldane's rule could result in the current scenario as well. Females are the heterogametic sex in birds, so Haldane's rule posits that female hybrids are more likely to be sterile than male hybrids, and this has been shown to be true (Price and Bouvier 2002). Assuming male hybrids among the clades are fully fertile, they would contribute to a loss of structure in biparentally inherited nuclear markers. If female hybrids were also fully fertile and there were no significant sex-biased dispersal, regions of mtDNA clade sympatry would closely mirror areas exhibiting loss of nuclear structure. However, if female hybrids exhibited reduced fertility according to Haldane's rule – which would not be unexpected at the level of genetic divergence present in *X*. *zosterops* (Price and Bouvier 2002) – the spread of mtDNA haplotypes would lag behind that of nuclear markers as mtDNA dispersal would be more reliant on nonhybrids. Distinguishing between these two possible scenarios – male-biased dispersal and Haldane's rule – to explain the lack of complete sympatry of *X*. *zosterops* mtDNA clades will require detailed field studies.

In combination, the data from *X*. *zosterops* mtDNA, microsatellites, and *Myrsidea* mtDNA reflect a widespread merger of previously allopatric lineages but do not provide information about the cause. To date, anthropogenic ecological changes, usually through habitat disturbance or introduction of non-native related species, have been invoked as the primary driving force behind examples of complete despeciation (e.g., Rhymer and Simberloff 1996; Seehausen et al. 1997, 2008; Seehausen 2006; Taylor et al. 2006; Vonlanthen et al. 2012; Hudson et al. 2013). Isolated lineages in their original, stable environment are likely to remain disjunct without such disturbances. Both natural changes in recent geological time and anthropogenic changes in the past few thousand years have affected Madagascar's humid forests and may have contributed to the merger of *X*. *zosterops* lineages.

Due to natural climate cycles associated with broad-scale global patterns in the Quaternary, humid forest habitats on Madagascar have periodically shifted in elevation (Straka 1996; Gasse and Van Campo 1998; Burney et al. 2004). These repeated altitudinal shifts resulted in waves of isolation and connection for various bird taxa between ecological habitats and different massifs (Raherilalao and Goodman 2011). Given these habitat shifts and considering that *X*. *zosterops* is sensitive to forest fragmentation (Langrand and Wilmé 1997; Raherilalao 2001), the current scenario could be the result of habitat fragmentation sometime after the late Tertiary followed by relatively recent reconnection and secondary contact. This hypothesis may be supported by the signature of demographic expansion in the mtDNA clades and data showing that humid forests significantly expanded from lower elevations beginning approximately 9800 years ago due to warming temperatures (Straka 1996; Gasse and Van Campo 1998; Burney et al. 2004).

Anthropogenic change, particularly in the past century, also has strongly affected current humid forest distribution on Madagascar. Widespread deforestation has resulted in the loss of ∼50% of the island's humid forests that existed in the 1950s (Harper et al. 2008). Deforestation at low altitudes has been particularly prevalent south of 17°S, where the sympatry of *X*. *zosterops* mtDNA clades is concentrated. This loss of habitat may have forced low-elevation *X*. *zosterops* populations upslope, leading to the current widespread sympatry of the clades and loss of structure in microsatellites, which can occur in less than ten generations between species without intrinsic barriers to hybridization (Taylor et al. 2006; Gilman and Behm 2011). However, without further data – ideally from samples predating the majority of the deforestation – the cause of the potential despeciation in *X*. *zosterops*, whether natural or anthropogenic, is likely to remain ambiguous.

## Conclusion

In addition to contributing to the study of vertebrate speciation, particularly in birds, this work adds to limited data concerning avian phylogeography on Madagascar. The focus of most phylogeographic studies on Madagascar has been amphibians, reptiles, or mammals, with only four utilizing extensive population-level sampling in birds (Fuchs et al. 2007, 2013; Cruaud et al. 2011; Goodman et al. 2011). This study represents the densest and largest sampling yet presented for a Malagasy endemic bird and the first for a species within one of Madagascar's endemic avian radiations. Although bird communities show differences between lowland and montane forests, this differentiation between habitats has not been suggested to play a direct role in bird diversification on the island. Studies on other vertebrates have highlighted cryptic taxa geographically split by elevation in humid forests (e.g., Olson et al. 2004), but *X*. *zosterops* provides the first example of this biogeographic pattern in birds.

As well as being informative about avian biogeography in Madagascar, this study illustrates the importance of lowland humid forest to preserving extant biodiversity on the island. This has been highlighted in other vertebrate taxa, but it has been underemphasized in birds. Low-elevation forests in the central east and southeast have not received attention as a center of endemism in birds, but the *X*. *zosterops* results show their importance. Further phylogeographic studies on Malagasy birds may yet uncover similar patterns and unknown cryptic taxa.

Finally, we highlight the value of gathering multiple types of data in phylogeographic studies to contribute to the study of vertebrate speciation. In this instance, data from mtDNA, nuclear DNA, and ectoparasites were necessary to better understand the complicated evolutionary history of *X*. *zosterops*. We also emphasize the importance of sampling across a taxon's distribution to provide greater insight into the biodiversity of a region and to reveal unknown patterns of regional endemism. These aspects contribute to more accurate and comprehensive biogeographic theories and provide important details for developing conservation programs.

## Supporting Information

Appendix S1. Materials and methods. **Table S1.** Genetic diversity statistics of eight microsatellite loci for each mtDNA clade of *X*. *zosterops*. **Table S2.** Pairwise genetic differentiation statistics for *X*. *zosterops* mtDNA clades, based on 8 microsatellite loci. **Figure S1.** Δ*K* values calculated from 10 Structure runs each of *K *=* *1–8. **Figure S2.** The average negative deviance information criterion (DIC) of the 10 lowest-DIC runs for each *K* in the TESS analyses plotted with the *H'* value for each *K* calculated in CLUMPP.

Table S3. List of all sequenced *Xanthomixis* specimens.

Table S4. List of all sequenced *Myrsidea* specimens.
