# Supplementary material for: Potential merger of ancient lineages in a passerine bird discovered based on evidence from host-specific ectoparasites
Source: Ecol Evol. 2015 Aug 18;5(17):3743–55. doi: 10.1002/ece3.1639 (PMC4567877; doi:10.1002/ece3.1639)
Supplement: Appendix S1. — Materials and methods. Table S1. Genetic diversity statistics of eight microsatellite loci for each mtDNA clade of X. zosterops. Table S2. Pairwise genetic differentiation statistics for X. zosterops mtDNA clades, based on 8 microsatellite loci. Figure S1. ΔK values calculated from 10 Structure runs each of K = 1–8. Figure S2. The average negative deviance information criterion (DIC) of the 10 lowest-DIC runs for each K in the TESS analyses plotted with the H' value for each K calculated in CLUMPP. [file ece30005-3743-sd1.docx]

**Supplemental Information**

**SI Materials and Methods**

**DNA Sequencing**

Primers L10755 and H11151 (Chesser, 1999) were used for ND3 amplification, L14990 (Kocher *et al.*, 1989) and H15916 (Edwards *et al.*, 1991) for cyt-*b*, and A8PWL and CO3HMH (G Seutin & E Bermingham, unpublished data) for ATP6. The polymerase chain reaction (PCR) conditions for ND3 followed this profile: 94°C for 2 min, 35 cycles of denaturation at 95°C for 15 sec, annealing at 60°C for 30 sec, extension at 72°C for 30 sec, and a final extension of 1 min. We used a similar profile for cyt-*b*, but the annealing step was at 56°C, the extension was for 1 min, and the final extension was for 2 min. For ATP6, the annealing step was at 52°C and the extension was for 50 sec.

For the *Myrsidea* samples, we used the primers L6625 and H7005 (Hafner *et al.*, 1994) to amplify COI. The following PCR profile was used for amplification of the COI fragment: 94°C for 10 min, 35 cycles of denaturation at 94°C for 30 sec, annealing at 46°C for 30 sec, extension at 65°C for 30 sec, and a final extension of 7 min.

Depending on amplification quality, we purified PCR products using an ExoSAP protocol or a GELase™ (Epicentre Technologies, Madison, WI) protocol. The purified products were cycle sequenced using BigDye® Terminator v3.1 chemistry (Applied Biosystems, Foster City, CA [AB]). We purified the cycle sequencing products using AB’s recommended ethanol/EDTA precipitation method, and the precipitated products were resuspended in Hi-Di™ Formamide and run on an AB 3730 DNA Analyzer.

**Phylogenetic analysis of sequence data**

Using the outputs from Kakusan4, we constructed a ML phylogeny with GARLI 2.0 (Zwickl, 2006), keeping the best tree of four independent searches and assessing nodal support with 1000 bootstrap replicates in RAxML (Stamatakis, 2006; Stamatakis *et al.*, 2008). We constructed a BI phylogeny with MrBayes 3.1.2 (Huelsenbeck & Ronquist, 2001; Ronquist & Huelsenbeck, 2003), with the following settings differing from the default MCMC settings: ngen=10,000,000, samplefreq=1000, burnin=2500. The ML and BI phylogenetic analyses were conducted on the CIPRES Science Gateway (Miller *et al.*, 2010). Tracer 1.5 (Rambaut & Drummond, 2009) was used to check for stationarity of the BI runs, and AWTY (Wilgenbusch *et al.*, 2004; Nylander *et al.*, 2008) was used to check for tree convergence. MP tree inference and nodal support were assessed via 1000 bootstrap replicates in PAUP* 4.0b10 (Swofford, 2002).

**Microsatellite genotyping**

Using the protocol of Glenn & Schable (2005) as a framework, we genotyped 10 novel, variable microsatellite loci for 104 individuals (Table S3). Five loci were amplified using a Type-It® Microsatellite PCR Kit (Qiagen, Valencia, CA) and dye-tagged primers. We altered the Type-It protocol to reduce the PCR reaction volume to 10 µL by using the following recipe: 3.5 µL H_2_O, 5.0 µL 2X Type-It Master Mix, 1.0 µL 10X primer mix, 0.5 µL of 50 ng/µL DNA template. The PCR thermal profile followed the Type-It protocol, using a 57°C annealing temperature.

We amplified the remaining five loci using standard PCR reactions and M13 primers. The 12.5-µL PCR recipe for these loci was 8.4 µL H_2_O, 1.25 µL GeneAmp® 10X PCR Buffer (Applied Biosystems), 0.5 µL 25mM MgCl_2_, 0.1 µL Taq DNA polymerase (Roche Applied Science, Indianapolis, IN), 0.5 µL bovine serum albumin (BSA), 0.1 µL 8mM dNTPs, 0.125 µL forward primer with M13 tail, 0.5 µL reverse primer, 0.5 µL dye-tagged M13 primer, and 0.5–1.2 µL DNA template. Amplification of these five loci followed this PCR profile: 94°C for 2 min, 34 cycles of denaturation at 95°C for 15 sec, annealing at 59°C for 30 sec, extension at 72°C for 30 sec, and a final extension of 2 min.

For genotyping, the samples were run an AB 3730 DNA Analyzer using the following recipe in each well: 0.5 µL PCR product, 9.28 µL Hi-Di™ Formamide, and 0.22 µL GeneScan™ –500 LIZ® Size Standard (Applied Biosystems). Peak Scanner™ Software v1.0 (Applied Biosystems) and STRand (Toonen & Hughes, 2001) were used to analyze the run results and to generate the genotypic data.

**Microsatellite analysis**

To increase the sensitivity of the Structure model to weak structure, sample location information was used as a prior. The number of clusters (*K*) modeled in the analysis was 1–8 to account for possible intraclade structure. The analysis was run for 1,000,000 MCMC replications, with 500,000 designated as burn-in. We performed 10 runs of the analysis to ensure the consistency of the results.

Structurama uses the same Bayesian clustering algorithm as Structure, but it can infer *K* by allowing it to vary following a Dirichlet process prior (Pella & Masuda, 2006; Huelsenbeck & Andolfatto, 2007). We ran Structurama for 10,000,000 generations with a 25% burn-in, allowing *K* and the expected prior *K* to vary. The program outputs the fraction of time that the MCMC run samples a partition (e.g. Pr(*K*=1)=0.80, Pr(*K*=2)=0.19). This is considered a valid approximation of the posterior probability of each partition, and the optimum *K* value is inferred to be the one with the highest probability.

For the TESS analyses, using the BYM model of admixture, we conducted 50 runs per *K* for *K*=2–8, each run comprising 4000 sweeps with a 50% burn-in. The 10 lowest-DIC runs for each *K* were summarized in CLUMPP.

**SI Results**

Two of the ten variable genotyped loci showed an excess of homozygotes and were not in Hardy-Weinberg equilibrium. Preliminary analyses of genetic differentiation and population structure showed that these two loci had a slight effect on genetic differentiation values (although population clustering conclusions were not affected). Thus, full analyses were conducted on a reduced dataset of eight loci. Two of the eight loci, despite being on different chromosomes according to a BLAST against the zebra finch genome, showed a weakly significant test for linkage disequilibrium. However, removal of one locus or both did not affect results, so final analyses used all eight loci. Gene diversity and allelic richness were uniformly high across all loci and clades (Table S1).

Structure’s result of *K*=1 also is supported by examination of α across the runs and by CLUMPP’s *H’* statistic. The α value during runs of *K*>1 often fails to stabilize, which reflects weak support for a given *K*. *H’*, which is very near 1.0 when there is strong support for a particular clustering pattern, shows a similar pattern of instability, with values in the range of 0.8–0.9 for *K*=2–8. Low values of *H’* also can reflect a multimodal likelihood surface for a particular *K*, but the results provide no evidence for a multimodal likelihood surface.

For the TESS analyses, negative DIC values increase as *K* increases from 2–8 (DIC cannot assess *K*=1), suggesting that the optimum *K* may be 8 (Fig. S2). However, accuracy of DIC conclusions decreases as *K* increases when associated with a high level of migration among populations (Gao *et al.*, 2011), which would be true if *K*=1. The inaccuracy of the DIC results is corroborated by CLUMPP’s *H’* statistic, which steadily decreases as *K* and DIC increase (Fig. S2). As with the Structure results, these low *H’* statistics are due to lack of convergence in population assignments rather than a multimodal likelihood surface.

Results from Structurama, which are clustering probabilities of Pr(*K*=1) = 0.9049 and Pr(*K*=2) = 0.0893, also support *K*=1. Values of genetic differentiation statistics among mtDNA clades are low (Table S2), and the AMOVA attributes 98.5% of variation to within individuals, 1.4% within clades, and 0.1% among clades. These results support a high level of migration among the clades.

**References**

Chesser, R.T. 1999. Molecular systematics of the rhinocryptid genus Pteroptochos. *Condor* **101**: 439–446.

Edwards, S. V., Arctander, P. & Wilson, A.C. 1991. Mitochondrial resolution of a deep branch in the genealogical tree for perching birds. *Proc. R. Soc. B Biol. Sci.* **243**: 99–107.

Gao, H., Bryc, K. & Bustamante, C.D. 2011. On identifying the optimal number of population clusters via the deviance information criterion. *PLoS One* **6**: e21014.

Glenn, T.C. & Schable, N.A. 2005. Isolating microsatellite DNA loci. *Methods Enzymol.* **395**: 202–222.

Hafner, M.S., Sudman, P.D., Villablanca, F.X., Spradling, T.A., Demastes, J.W. & Nadler, S.A. 1994. Disparate rates of molecular evolution in cospeciating hosts and parasites. *Science (80-. ).* **265**: 1087–1090.

Huelsenbeck, J.P. & Andolfatto, P. 2007. Inference of population structure under a Dirichlet process model. *Genetics* **175**: 1787–1802.

Huelsenbeck, J.P. & Ronquist, F. 2001. MRBAYES: Bayesian inference of phylogenetic trees. *Bioinformatics* **17**: 754–755.

Kocher, T.D., Thomas, W.K., Meyer, A., Edwards, S. V, Pääbo, S., Villablanca, F.X., *et al.* 1989. Dynamics of mitochondrial DNA evolution in animals: Amplification and sequencing with conserved primers. *Proc. Natl. Acad. Sci. U. S. A.* **86**: 6196–6200.

Miller, M.A., Pfeiffer, W. & Schwartz, T. 2010. Creating the CIPRES Science Gateway for inference of large phylogenetic trees. In: *Proceedings of the Gateway Computing Environments Workshop (GCE)*, pp. 1–8. New Orleans, Louisiana.

Nylander, J.A.A., Wilgenbusch, J.C., Warren, D.L. & Swofford, D.L. 2008. AWTY (are we there yet?): A system for graphical exploration of MCMC convergence in Bayesian phylogenetics. *Bioinformatics* **24**: 581–583.

Pella, J. & Masuda, M. 2006. The Gibbs and split merge sampler for population mixture analysis from genetic data with incomplete baselines. *Can. J. Fish. Aquat. Sci.* **63**: 576–596.

Rambaut, A. & Drummond, A.J. 2009. Tracer v1.5. http://tree.bio.ed.ac.uk/software/tracer/.

Ronquist, F. & Huelsenbeck, J.P. 2003. MrBayes 3: Bayesian phylogenetic inference under mixed models. *Bioinformatics* **19**: 1572–1574.

Stamatakis, A. 2006. RAxML-VI-HPC: Maximum likelihood-based phylogenetic analyses with thousands of taxa and mixed models. *Bioinformatics* **22**: 2688–2690.

Stamatakis, A., Hoover, P. & Rougemont, J. 2008. A rapid bootstrap algorithm for the RAxML web-servers. *Syst. Biol.* **75**: 758–771.

Swofford, D.L. 2002. *PAUP*: Phylogenetic Analysis Using Parsimony (and Other Methods), v. 4.0b10*. Sinauer Associates, Sunderland, Massachusetts.

Toonen, R. & Hughes, S. 2001. Increased throughput for fragment analysis on an ABI PRISM 377 automated sequencer using a membrane comb and STRand software. *Biotechniques* **31**: 1320–1324.

Wilgenbusch, J.C., Warren, D.L. & Swofford, D.L. 2004. AWTY: A system for graphical exploration of MCMC convergence in Bayesian phylogenetic inference. http://ceb.csit.fsu.edu/awty.

Zwickl, D.J. 2006. Genetic algorithm approaches for the phylogenetic analysis of large biological sequence datasets under the maximum likelihood criterion. University of Texas, Austin, TX. Available from http://garli.googlecode.com.

**Table S1.** Genetic diversity statistics of eight microsatellite loci for each mtDNA clade of *X*. *zosterops*.

| **Locus** | **# of Alleles** | | | |  | **Gene Diversity** | | | |  | **Allelic Richness** | | | |
| --- | --- | --- | --- | --- | --- | --- | --- | --- | --- | --- | --- | --- | --- | --- |
|  | **1** | **2** | **3** | **4** |  | **1** | **2** | **3** | **4** |  | **1** | **2** | **3** | **4** |
| **xz3453.02** | 11 | 14 | 12 | 12 |  | 0.88 | 0.91 | 0.90 | 0.91 |  | 9.60 | 11.53 | 12.00 | 11.29 |
| **xz3453.05** | 15 | 15 | 12 | 13 |  | 0.89 | 0.90 | 0.89 | 0.88 |  | 11.21 | 11.50 | 12.00 | 11.33 |
| **xz3453.06** | 14 | 12 | 11 | 10 |  | 0.88 | 0.89 | 0.89 | 0.89 |  | 11.13 | 10.10 | 11.00 | 9.46 |
| **xz3453.12** | 13 | 12 | 11 | 10 |  | 0.92 | 0.89 | 0.92 | 0.89 |  | 11.29 | 10.64 | 11.00 | 9.75 |
| **xz3453.16** | 16 | 18 | 14 | 17 |  | 0.93 | 0.93 | 0.94 | 0.93 |  | 12.90 | 13.22 | 14.00 | 14.62 |
| **xz3453.20** | 11 | 18 | 13 | 12 |  | 0.91 | 0.91 | 0.91 | 0.90 |  | 10.07 | 12.53 | 13.00 | 10.91 |
| **xz3453.22** | 16 | 15 | 12 | 15 |  | 0.91 | 0.93 | 0.91 | 0.92 |  | 12.31 | 12.69 | 12.00 | 13.13 |
| **xz8608.01** | 12 | 12 | 11 | 10 |  | 0.88 | 0.87 | 0.91 | 0.89 |  | 10.08 | 9.67 | 11.00 | 9.57 |

**Table S2.** Pairwise genetic differentiation statistics for *X*. *zosterops* mtDNA clades, based on 8 microsatellite loci. Test values are below the diagonals, and p-values are above. P-values have been adjusted using the Benjamini & Hochberg (1995) step-up false discovery rate controlling procedure.

| **mtDNA Clade** | ***F_ST_*** | | | |  | ***R_ST_*** | | | |  | **Jost’s D** | | | | |
| --- | --- | --- | --- | --- | --- | --- | --- | --- | --- | --- | --- | --- | --- | --- | --- |
|  | **1** | **2** | **3** | **4** |  | **1** | **2** | **3** | **4** |  | **1** | **2** | **3** | **4** |  |
| **1** | - | 0.32 | 0.66 | 0.64 |  | - | 0.19 | 0.19 | 0.34 |  | - |  |  |  |  |
| **2** | 0.004 | - | 0.32 | 0.82 |  | 0.015 | - | 0.34 | 0.40 |  | 0.005 | - |  |  |  |
| **3** | 0.001 | 0.005 | - | 0.82 |  | 0.031 | 0.006 | - | 0.34 |  | 0.000 | 0.018 | - |  |  |
| **4** | 0.001 | 0.000 | 0.000 | - |  | 0.005 | 0.000 | 0.005 | - |  | 0.001 | 0.000 | 0.000 | - |  |

Figure S1**.** Δ*K* values calculated from 10 Structure runs each of *K*=1–8.

Figure S2**.** The average negative deviance information criterion (DIC) of the 10 lowest-DIC runs for each *K* in the TESS analyses plotted with the *H’* value for each *K* calculated in CLUMPP.
