# Supplementary material for: Potential merger of ancient lineages in a passerine bird discovered based on evidence from host-specific ectoparasites
Source: Ecol Evol. 2015 Aug 18;5(17):3743–55. doi: 10.1002/ece3.1639 (PMC4567877; doi:10.1002/ece3.1639)
Supplement: Table S3. — List of all sequenced Xanthomixis specimens. [file ece30005-3743-sd2.docx]

**Table S3.** List of all sequenced *Xanthomixis* specimens.

| **Species** | **Voucher #** | **mtDNA Clade** | **Micro-satellites** | **GenBank Accession Numbers** | | |
| --- | --- | --- | --- | --- | --- | --- |
|  |  |  |  | **cyt-*b*** | **ND3** | **ATP** |
| *X. apperti* | FMNH 393159 | - | - | KT313824 | KT313941 |  |
| *X. cinereiceps* | FMNH 363829 | - | - | KT313825 | KT313942 |  |
| *X. zosterops* | FMNH 345743 | 4 | X | KT313826 | KT313943 | KT313771 |
| *X. zosterops* | FMNH 345744 | 4 | X | KT313827 | KT313944 | KT313772 |
| *X. zosterops* | FMNH 345746 | 2 | X | KT313828 | KT313945 | KT313773 |
| *X. zosterops* | FMNH 345747 | 2 | X | KT313829 | KT313946 | KT313774 |
| *X. zosterops* | FMNH 345749 | 3 | X | KT313830 | KT313947 | KT313775 |
| *X. zosterops* | FMNH 345750 | 4 | X | KT313831 | KT313948 | KT313776 |
| *X. zosterops* | FMNH 345752 | 4 | X | KT313832 | KT313949 | KT313777 |
| *X. zosterops* | FMNH 345754 | 3 | X | KT313833 | KT313950 | KT313778 |
| *X. zosterops* | FMNH 345758 | 4 | X | KT313834 | KT313951 | KT313779 |
| *X. zosterops* | FMNH 356645 | 1 | X | KT313835 | KT313952 | KT313780 |
| *X. zosterops* | FMNH 356646 | 1 | X | KT313836 | KT313953 | KT313781 |
| *X. zosterops* | FMNH 356647 | 1 | X | KT313837 | KT313954 | KT313782 |
| *X. zosterops* | FMNH 363820 | 4 | X | KT313838 | KT313955 | KT313783 |
| *X. zosterops* | FMNH 363824 | 2 | X | KT313839 | KT313956 | KT313784 |
| *X. zosterops* | FMNH 384740 | 1 | X | KT313840 | KT313957 | KT313785 |
| *X. zosterops* | FMNH 393137 | 2 | X | KT313841 | KT313958 | KT313786 |
| *X. zosterops* | FMNH 393154 | 2 | X | KT313842 | KT313959 | KT313787 |
| *X. zosterops* | FMNH 393265 | 1 | X | KT313843 | KT313960 | KT313788 |
| *X. zosterops* | FMNH 393266 | 1 | X | KT313844 | KT313961 | KT313789 |
| *X. zosterops* | FMNH 393269 | 1 | X | KT313845 | KT313962 | KT313790 |
| *X. zosterops* | FMNH 393270 | 1 | X | KT313846 | KT313963 | KT313791 |
| *X. zosterops* | FMNH 393271 | 1 | X | KT313847 | KT313964 | KT313792 |
| *X. zosterops* | FMNH 393272 | 1 | X | KT313848 | KT313965 | KT313793 |
| *X. zosterops* | FMNH 393273 | 1 | X | KT313849 | KT313966 | KT313794 |
| *X. zosterops* | FMNH 393275 | 4 | X | KT313850 | KT313967 | KT313795 |
| *X. zosterops* | FMNH 393277 | 2 | X | KT313851 | KT313968 | KT313796 |
| *X. zosterops* | FMNH 396141 | 4 | X | KT313852 | KT313969 | KT313797 |
| *X. zosterops* | FMNH 396143 | 1 | X | KT313853 | KT313970 | KT313798 |
| *X. zosterops* | FMNH 396144 | 1 | X | KT313854 | KT313971 | KT313799 |
| *X. zosterops* | FMNH 396149 | 1 | X | KT313855 | KT313972 | KT313800 |
| *X. zosterops* | FMNH 396151 | 1 | X | KT313856 | KT313973 | KT313801 |
| *X. zosterops* | FMNH 427370 | 2 | X | KT313857 | KT313974 |  |
| *X. zosterops* | FMNH 427374 | 3 | X | KT313858 | KT313975 |  |
| *X. zosterops* | FMNH 431197 | 1 | X | KT313859 | KT313976 | KT313802 |
| *X. zosterops* | FMNH 431215 | 1 | X | KT313860 | KT313977 |  |
| *X. zosterops* | FMNH 431216 | 1 | X | KT313861 | KT313978 |  |
| *X. zosterops* | FMNH 431217 | 1 | X | KT313862 | KT313979 |  |
| *X. zosterops* | FMNH 431218 | 1 | X | KT313863 | KT313980 |  |
| *X. zosterops* | FMNH 431219 | 1 | X | KT313864 | KT313981 |  |
| *X. zosterops* | FMNH 431220 | 1 | X | KT313865 | KT313982 |  |
| *X. zosterops* | FMNH 431221 | 1 | X | KT313866 | KT313983 |  |
| *X. zosterops* | FMNH 434656 | 1 |  | KT313867 | KT313984 |  |
| *X. zosterops* | FMNH 438696 | 4 | X | KT313868 | KT313985 | KT313803 |
| *X. zosterops* | FMNH 438697 | 2 | X | KT313869 | KT313986 | KT313804 |
| *X. zosterops* | FMNH 438698 | 3 | X | KT313870 | KT313987 | KT313805 |
| *X. zosterops* | FMNH 438700 | 3 | X | KT313871 | KT313988 | KT313806 |
| *X. zosterops* | FMNH 438701 | 4 | X | KT313872 | KT313989 | KT313807 |
| *X. zosterops* | FMNH 438702 | 4 | X | KT313873 | KT313990 | KT313808 |
| *X. zosterops* | FMNH 438704 | 2 | X | KT313874 | KT313991 |  |
| *X. zosterops* | FMNH 479565 | 3 | X | KT313875 | KT313992 |  |
| *X. zosterops* | FMNH 479566 | 2 | X | KT313876 | KT313993 |  |
| *X. zosterops* | FMNH 479567 | 2 |  | KT313877 | KT313994 |  |
| *X. zosterops* | FMNH 479568 | 2 |  | KT313878 | KT313995 |  |
| *X. zosterops* | FMNH 479569 | 4 | X | KT313879 | KT313996 |  |
| *X. zosterops* | FMNH 479570 | 4 | X | KT313880 | KT313997 |  |
| *X. zosterops* | FMNH 479571 | 4 |  | KT313881 | KT313998 |  |
| *X. zosterops* | FMNH 479572 | 3 | X | KT313882 | KT313999 |  |
| *X. zosterops* | FMNH 479573 | 3 | X | KT313883 |  |  |
| *X. zosterops* | FMNH 479574 | 3 | X | KT313884 |  |  |
| *X. zosterops* | FMNH 479575 | 3 | X | KT313885 |  |  |
| *X. zosterops* | FMNH 479576 | 4 | X | KT313886 | KT314000 |  |
| *X. zosterops* | FMNH 479577 | 3 | X | KT313887 | KT314001 |  |
| *X. zosterops* | FMNH 479578 | 4 |  | KT313888 | KT314002 |  |
| *X. zosterops* | FMNH 479579 | 4 |  | KT313889 | KT314003 |  |
| *X. zosterops* | FMNH 479580 | 4 |  | KT313890 | KT314004 |  |
| *X. zosterops* | FMNH 479581 | 4 |  | KT313891 | KT314005 |  |
| *X. zosterops* | FMNH 479582 | 4 |  | KT313892 | KT314006 |  |
| *X. zosterops* | FMNH 479583 | 4 | X | KT313893 | KT314007 |  |
| *X. zosterops* | FMNH 479584 | 4 | X | KT313894 | KT314008 |  |
| *X. zosterops* | FMNH 479585 | 4 | X | KT313895 | KT314009 |  |
| *X. zosterops* | FMNH 479587 | 4 |  | KT313896 | KT314010 |  |
| *X. zosterops* | FMNH 479588 | 2 | X | KT313897 |  |  |
| *X. zosterops* | FMNH 479589 | 2 | X | KT313898 | KT314011 |  |
| *X. zosterops* | FMNH 479590 | 2 | X | KT313899 | KT314012 |  |
| *X. zosterops* | FMNH 479643 | 2 |  | KT313900 |  |  |
| *X. zosterops* | FMNH 479644 | 2 |  | KT313901 | KT314013 | KT313809 |
| *X. zosterops* | FMNH 479645 | 2 |  | KT313902 | KT314014 | KT313810 |
| *X. zosterops* | UADBA 10869 | 3 | X | KT313903 | KT314015 | KT313811 |
| *X. zosterops* | UADBA 10969 | 1 | X | KT313904 | KT314016 | KT313812 |
| *X. zosterops* | UADBA 10970 | 1 | X | KT313905 | KT314017 |  |
| *X. zosterops* | UADBA 10995 | 1 | X | KT313906 | KT314018 |  |
| *X. zosterops* | UADBA 30900 | 2 | X | KT313907 | KT314019 |  |
| *X. zosterops* | UADBA 30917 | 2 | X | KT313908 | KT314020 |  |
| *X. zosterops* | UADBA 30929 | 2 | X | KT313909 | KT314021 |  |
| *X. zosterops* | UADBA 30939 | 2 | X | KT313910 | KT314022 |  |
| *X. zosterops* | UADBA 30943 | 2 | X | KT313911 | KT314023 |  |
| *X. zosterops* | UADBA 30953 | 2 | X | KT313912 | KT314024 |  |
| *X. zosterops* | UADBA 30956 | 2 | X | KT313913 |  |  |
| *X. zosterops* | UADBA 30966 | 2 | X | KT313914 | KT314025 |  |
| *X. zosterops* | UADBA 31305 | 2 | X | KT313915 | KT314026 |  |
| *X. zosterops* | UADBA 31307 | 2 | X | KT313916 |  |  |
| *X. zosterops* | UADBA 31308 | 4 | X | KT313917 | KT314027 | KT313813 |
| *X. zosterops* | UADBA 31312 | 2 | X | KT313918 | KT314028 | KT313814 |
| *X. zosterops* | UADBA 47215 | 1 | X | KT313919 | KT314029 | KT313815 |
| *X. zosterops* | UADBA 47216 | 1 | X | KT313920 | KT314030 | KT313816 |
| *X. zosterops* | UADBA 47217 | 1 | X | KT313921 | KT314031 | KT313817 |
| *X. zosterops* | UADBA 47218 | 2 | X | KT313922 | KT314032 | KT313818 |
| *X. zosterops* | UADBA 47220 | 3 | X | KT313923 | KT314033 | KT313819 |
| *X. zosterops* | UADBA 47221 | 3 | X | KT313924 | KT314034 | KT313820 |
| *X. zosterops* | UADBA 47222 | 4 | X | KT313925 | KT314035 |  |
| *X. zosterops* | UADBA 47223 | 3 | X | KT313926 | KT314036 |  |
| *X. zosterops* | UADBA 47720 | 2 | X | KT313933 | KT314045 |  |
| *X. zosterops* | UADBA 47721 | 2 | X | KT313934 | KT314046 |  |
| *X. zosterops* | UADBA MJR68 | 2 | X | KT313935 | KT314047 |  |
| *X. zosterops* | UADBA MJR77 | 2 | X | KT313927 | KT314037 |  |
| *X. zosterops* | UADBA MJR84 | 2 | X | KT313928 | KT314038 |  |
| *X. zosterops* | UADBA MJR121 | 2 | X | KT313929 | KT314039 |  |
| *X. zosterops* | UADBA MJR122 | 2 | X | KT313930 | KT314040 |  |
| *X. zosterops* | UADBA MJR126 | 2 | X | KT313931 | KT314041 |  |
| *X. zosterops* | UADBA MJR127 | 2 | X | KT313932 | KT314042 |  |
| *X. zosterops* | UADBA MJR133 | 2 | X |  | KT314043 |  |
| *X. zosterops* | UADBA MJR135 | 2 | X |  | KT314044 |  |
| *X. zosterops* | UADBA NLB01 | 4 | X | KT313936 | KT314048 |  |
| *X. zosterops* | UADBA SMG8608 | 2 | X | KT313939 | KT314051 | KT313822 |
| *X. zosterops* | UADBA SMG10703 | 1 | X | KT313937 | KT314049 | KT313821 |
| *X. zosterops* | UADBA SMG16187 | 1 | X | KT313938 | KT314050 |  |
| *X. zosterops* | UADBA TPG1562 | 2 | X | KT313940 | KT314052 | KT313823 |
