# Supplementary material for: Potential merger of ancient lineages in a passerine bird discovered based on evidence from host-specific ectoparasites
Source: Ecol Evol. 2015 Aug 18;5(17):3743–55. doi: 10.1002/ece3.1639 (PMC4567877; doi:10.1002/ece3.1639)
Supplement: Table S4. — List of all sequenced Myrsidea specimens. [file ece30005-3743-sd3.docx]

**Table S4.** List of all sequenced *Myrsidea* specimens.

| **Species/Phylogroup** | **FMNH-INS#** | **Voucher #** | **Host Species** | **Host FMNH #** | **Host mtDNA Clade** | **COI GenBank Accession Numbers** |
| --- | --- | --- | --- | --- | --- | --- |
| *Myrsidea* sp. ex *Crossleyia xanthophrys* | 29126 | My.CX.5.11.2011.22 | *Crossleyia xanthophrys* | 479640 | - | KT314053 |
| *Myrsidea yoshizawai* | 28493 | Mysp.Phzos.8.16.2005.4 | *Xanthomixis zosterops* | 431219 | 1 | DQ860181.1 |
| *Myrsidea* sp. B | 29105 | My.XZ.5.11.2011.1 | *Xanthomixis zosterops* | 479566 | 2 | KT314054 |
| *Myrsidea* sp. B | 29106 | My.XZ.5.11.2011.2 | *Xanthomixis zosterops* | 479569 | 4 | KT314055 |
| *Myrsidea* sp. B | 29107 | My.XZ.5.11.2011.3 | *Xanthomixis zosterops* | 479570 | 4 | KT314056 |
| *Myrsidea* sp. A | 29108 | My.XZ.5.11.2011.4 | *Xanthomixis zosterops* | 479571 | 4 | KT314057 |
| *Myrsidea* sp. A | 29109 | My.XZ.5.11.2011.5 | *Xanthomixis zosterops* | 479572 | 3 | KT314058 |
| *Myrsidea* sp. A | 29110 | My.XZ.5.11.2011.6 | *Xanthomixis zosterops* | 479573 | 3 | KT314059 |
| *Myrsidea* sp. A | 29111 | My.XZ.5.11.2011.7 | *Xanthomixis zosterops* | 479575 | 3 | KT314060 |
| *Myrsidea* sp. A | 29112 | My.XZ.5.11.2011.8 | *Xanthomixis zosterops* | 479576 | 4 | KT314061 |
| *Myrsidea* sp. A | 29113 | My.XZ.5.11.2011.9 | *Xanthomixis zosterops* | 479577 | 3 | KT314062 |
| *Myrsidea* sp. A | 29114 | My.XZ.5.11.2011.10 | *Xanthomixis zosterops* | 479580 | 4 | KT314063 |
| *Myrsidea* sp. A | 29115 | My.XZ.5.11.2011.11 | *Xanthomixis zosterops* | 479582 | 4 | KT314064 |
| *Myrsidea* sp. A | 29116 | My.XZ.5.11.2011.12 | *Xanthomixis zosterops* | 479583 | 4 | KT314065 |
| *Myrsidea* sp. A | 29117 | My.XZ.5.11.2011.13 | *Xanthomixis zosterops* | 479585 | 4 | KT314066 |
| *Myrsidea* sp. A | 29118 | My.XZ.5.11.2011.14 | *Xanthomixis zosterops* | 479587 | 4 | KT314067 |
| *Myrsidea* sp. B | 29119 | My.XZ.5.11.2011.15 | *Xanthomixis zosterops* | 479588 | 2 | KT314068 |
| *Myrsidea* sp. B | 29120 | My.XZ.5.11.2011.16 | *Xanthomixis zosterops* | 479589 | 2 | KT314069 |
| *Myrsidea* sp. B | 29121 | My.XZ.5.11.2011.17 | *Xanthomixis zosterops* | 479590 | 2 | KT314070 |
